# Functional Variation in Dipteran Gut Bacterial Communities in Relation to Their Diet, Life Cycle Stage and Habitat

**DOI:** 10.3390/insects11080543

**Published:** 2020-08-17

**Authors:** Rebekka Sontowski, Nicole M. van Dam

**Affiliations:** 1 German Centre for Integrative Biodiversity Research (iDiv) Halle-Jena-Leipzig, Deutscher Platz 5e, 04103 Leipzig, Germany; nicole.vandam@idiv.de; 2 Institute for Biodiversity, Friedrich-Schiller University, Dornburger Str. 159, 07743 Jena, Germany

**Keywords:** bacteria, insect–microbe interaction, host symbiosis, development, food source, malaria, pathogen vectors, pest management

## Abstract

**Simple Summary:**

Like in many other organisms, the guts of insects are full with many different bacteria. These bacteria can help their hosts to overcome toxic diets or can boost their resistance to pathogens. We were curious to learn which factors determine the composition of gut bacterial communities (GBCs) in true flies and mosquitoes, which belong to the order Diptera. We searched for research papers reporting on GBCs in these insects. Using these published data, we investigated whether the GBCs are species-specific, or whether they are determined by the diet, life stage or environment of the host insect. We found that the GBCs in larvae and adults of the same insect species can be very different. Insects on similar diets did not necessarily show similar GBCs. This made us conclude that GBCs are mostly life stage-specific. However, we found that the number of data papers we could use is limited; more data are needed to strengthen our conclusion. Lastly, novel DNA technologies can show ‘who is there’ in GBCs. At the same time, we lack knowledge on the exact function of gut bacteria. Obtaining more knowledge on the function of GBCs may help to design sustainable pest control measures.

**Abstract:**

True flies and mosquitos (Diptera) live in habitats and consume diets that pose specific demands on their gut bacterial communities (GBCs). Due to diet specializations, dipterans may have highly diverse and species-specific GBCs. Dipterans are also confronted with changes in habitat and food sources over their lifetime, especially during life history processes (molting, metamorphosis). This may prevent the development of a constant species- or diet-specific GBC. Some dipterans are vectors of several human pathogens (e.g., malaria), which interact with GBCs. In this review, we explore the dynamics that shape GBC composition in some Diptera species on the basis of published datasets of GBCs. We thereby focus on the effects of diet, habitats, and life cycle stages as sources of variation in GBC composition. The GBCs reported were more stage-specific than species- or diet-specific. Even though the presence of GBCs has a large impact on the performance of their hosts, the exact functions of GBCs and their interactions with other organisms are still largely unknown, mainly due to the low number of studies to date. Increasing our knowledge on dipteran GBCs will help to design pest management strategies for the reduction of insecticide resistance, as well as for human pathogen control.

## 1. Introduction

True flies and mosquitos (Diptera) are both a blessing and a curse for humans. On the one hand, several dipterans, such as hoverflies (Syrphidae) and humbleflies (Bombyliidae), fulfil important ecosystem services as pollinators [1,2]. On the other hand, some species are agricultural pests or vectors of human diseases, such as the Mediterranean fruit fly (Tephritidae) or the tsetse fly (Hippoboscoidae) [3,4]. Diptera is one of the most species-rich insect orders with an estimated number of 155,000 species spread around the world [5]. The large number of species and ecological functions emerged because dipterans adapted to a wide range of ecological niches. To exploit a wide diversity of niches, various species adapted to harsh and noxious environments or food sources that are inaccessible to other species [6,7]. This led to the emergence of many feeding strategies within this large insect order, which contains specialist feeding on plant tissues, pollen, nectar, vertebrate tissues, blood, carrion, feces, or other invertebrates. Each of these specialists face specific challenges with regards to the digestibility and detoxification of their diets.

To understand adaptations of Diptera to specific niches and diets, we should also consider their microbiomes, which mainly comprise bacteria. This consortium of microorganisms is an intrinsic part of the “holobiont”. A holobiont includes the host organism and all associated microorganisms and their interactions [8]. Microbes can be mutualists (of both host and microbe benefit), parasites (harmful to host), or commensals (neutral to host). The outcomes of host–microbe interactions shape the composition of microbial communities in hosts and can shape hosts’ performance [9,10]. Host performance can be particularly enhanced when their gut microbiome supports nutrient acquisition and the detoxification of diets [11,12].

Gut microbiomes include all bacteria, fungi, protists, and viruses living in the digestive tract of macroorganisms. In this review, we focus on bacteria and refer to them as gut bacterial communities (GBCs). GBCs provide nutrients, digest recalcitrant food sources, detoxify noxious compounds in the diet, and fend off harmful organisms [13,14,15]. On the basis of the high rate of food specialization within the Diptera, we expect a mutual adaptation between hosts and their gut microbiome. This mutual adaptation would result in a species-specific and stable GBC, especially in specialists. However, Diptera are holometabolic insects; in other words, they go through distinct developmental stages including complete metamorphosis in the pupal stage. Complete metamorphosis is frequently coupled with diet shifts and changes in environmental conditions. Mosquito larvae, for example, live in aquatic habitats and feed on algae, whereas the adults live in terrestrial habitats and feed on nectar and, in females only, on blood [16,17]. These changes pose specific requirements to GBCs and likely result in a variable GBC composition over the life cycle of a single insect [18]. In addition to the composition, GBC diversity may also be of importance. A highly diverse GBC colonization of the gut may help dipterans to meet the different requirements posed by different life stages as well as help them to adapt to a wide range of (variable) environments. A high GBC diversity may be especially advantageous for generalists, which are confronted with a broad range of food sources and toxins. Studies on GBC in another order of holometabolic insects, the lepidoptera, presented controversial results. Some bacteria seem to be diet-specific [19], whereas other studies revealed no correlation with food sources [20]. Finally, dipterans are often vectors for vertebrate pathogens, for instance pathogens that cause malaria or dengue fever [21,22]. These vertebrate pathogens may shift GBC composition and density [23].

In this review, we report the current status quo and explore the relevance of variation in GBCs in Diptera. Using published datasets, we examine to what extent we can draw conclusions about species-specific GBCs. In addition, GBCs may vary within single dipteran species over different life stages, which come with shifts in diet or environmental conditions. Additionally, we will discuss the importance and effects of single bacterial species on GBC composition and the interactions of dipteran GBCs with human pathogens.

## 2. GBCs Are Supporting Diptera to Exploit Specific Niches and Food Sources

Dipterans exploit a wide variety of diets, including plant tissues (such as pollen and nectar), vertebrate tissues, blood, feces, carrion, and invertebrates. These diets range from nearly sterile, such as blood, to diets that support enriched microbial communities, such as carrion or feces. At the same time, dipteran diets may contain chemical defenses, antibiotics, or indigestible compounds. Dipterans feeding on living hosts, such as herbivores, mosquitos, or predators, will be confronted with the immune responses of their host. The utilization of recalcitrant resources and the colonization of these specific niches require special adaptations. Establishing interactions with pre-adapted microorganisms will facilitate the adaptation process. Microbes have occupied a large variety of exceptional habitats for millions of years before macroorganisms started to colonize our planet [24]. Dipterans successfully acquired some of these microbes, most notably of the genera *Wolbachia* and *Wigglesworthia*, as symbionts [25,26,27,28] These endosymbionts can affect host reproduction, immunity, nutrient status and fitness, including the sex ratio of the offspring [9,25,29]. It is estimated that ancestors of tsetse flies were already infected with *Wigglesworthia glossinidia*-related bacteria approximately 50–100 million years ago [13]. *Wolbachia* infections in *Drosophila simulans* and *Culex pipiens* are dated about 58–66 million years ago [30]. In addition to these endosymbionts, insects carry a rich variety of other microbes inside and outside their bodies. A hotspot of microbes in insect bodies is their gut. Gut microbes provide important metabolic functions, including the digestion of food, the synthesis of micronutrients, and the conversion of recalcitrant resources, such as polysaccharides, to carbohydrates that can be absorbed by the gut [31]. For instance, the olive fruit fly *Bactrocera oleae* (Rossi) is a specialist on olive fruits. Olives contain a high proportion of indigestible proteins [32]. The gut bacterium *Pseudomonas savastanoi* hydrolyses the indigestible proteins in olive fruit flesh and converts them to amino acids [12]. After removing the GBCs via antibiotic treatments from the olive fruit fly adults, the larvae failed to reach the pupal stage in unripe olive fruits [12,33]. The “sterile” larvae were unable to digest unripe olives or non-hydrolyzed proteins [12]. Hagen [12] suggested that *P. savastanoi* hydrolyses the olive proteins and synthesizes the amino acids threonine and methionine. Both amino acids were not detected in the olive fruits but are essential for host development. In general, unripe fruits contain low levels of nutrients. This makes unripe fruits unattractive for herbivores. Olive fruit flies solve this challenge through a symbiotic interaction with the bacterium *Candidatus* Erwinia dacicola. This beneficial bacterium either directly serves as an amino acid and protein source, or it increases the protein digestibility in unripe olives [33]. Another fruit fly, the Mediterranean fruit fly, or Medfly, *Ceratitis capitata* (Wiedemann), is unable to acquire sufficient nitrogen from its diet, which mainly comprises soft fruits. This fly established symbioses with several nitrogen-fixing gut microbes that provide nitrogen to their host [34].

Next to recalcitrant resources, several insects are confronted with toxic compounds in their environments and/or diets, especially when they are confronted with novel or changed environments. GBCs are able to support some hosts through the detoxification of plant allelochemicals, pesticides, drugs, and reactive oxygen species (ROS). For instance, the larvae of the Chironomid *Chironomus javanus* can live in heavy metal-contaminated environments. *Ch. javanus* larvae produce the enzymes glutathione S-transferase (GST) and metallothionein (MT) [35]. GSTs make heavy metals less toxic by making them more water-soluble. This enables the larvae to excrete the heavy metals quickly [35]. MTs bind several metals and transport them to the cytosol. There they are stored separately from fundamental organelles. This process prevents essential organelles from being damaged by the heavy metals [36]. Another study showed that chironomid larvae harbor several adapted bacteria in their guts (e.g., *Shewanella decolorationis*, *Chromobacterium aquaticum*), which are able to detoxify lead and chromium [37]. The authors also found that larvae without the bacteria *S. decolorationis* or *C. aquaticum* had a lower survival rate in heavy metal environments compared with those with these bacteria. This suggest that these bacteria protect the chironomid larvae against toxic metals [37]. The fact that specific bacteria can improve survival in heavy metal environments may indicate that the GSTs and MTs needed for the detoxification are provided by these bacteria. Herbivorous dipterans have to deal with other natural toxins; plant tissues commonly contain plant defense compounds, which the insects ingest when feeding. GBCs can be confronted with these compounds, depending on the gut structure of the insect and their spatial distribution in the gut (foregut, midgut, or hindgut). Several studies showed that members of GBCs possess detoxification mechanisms, which may be beneficial to the GBCs as well as their hosts [15,38].

One of the oldest examples of gut microbes detoxifying defense compounds in their host’s diet was reported in olive fruit flies ([12]). Next to the indigestible proteins mentioned above, young olive fruits contain bitter-tasting defense allelochemicals, such as the phenolic compound oleuropein [32]. This makes them unpalatable to humans unless they are curated in water or brine. Experimental studies showed that oleuropein and derivatives thereof can deter herbivores, including ovipositing female olive fruit flies [39]. Oleuropein also reduces the availability of dietary proteins and lysine in unripe olives. Unripe olives in particular contain a high amount of oleuropein [33]. That makes unripe olives unpalatable for most insects. The olive fruit fly larvae can still feed on these fruits because they form a symbiotic relationship with their gut bacterium Ca. Erwinia dacicola. It is not exactly known how these bacteria overcome the effects of oleuropin, which also has antimicrobial and antifungal effects [40,41]. Presumably, this beneficial microbe degrades or binds oleuropein, or it uses enzymes resistant to the inhibitory effects of oleuropein to digest unripe olives [33].

The cabbage root fly (*Delia radicum* L.) is another example of an insect herbivore hosting GBCs that can detoxify plant defenses. Larvae of *D. radicum* are specialists in feeding on roots of species in the family Brassicaceae [42]. Just like the leaves, the roots contain glucosinolates, a class of anti-herbivore defense compounds typical for the Brassicaceae [43]. Upon damage, for example by *D. radicum* larvae, glucosinolates are mixed with the enzyme myrosinase. This causes the conversion of the glucosinolates in several breakdown products, including the toxic and deterrent isothiocyanates [44]. The glucosinolate 2-phenylethyl is one of the prominent glucosinolates in the roots of Brassicacea [45]. When root flies are feeding on the plant, phenylethyl-isothiocyanate is formed [46,47]. Several Gammaproteobacteria that can detoxify phenylethyl-isothiocyanate were isolated from the guts of cabbage root fly larvae [48]. Several of these bacteria possess a *saxA* gene encoding for a hydrolase that can break down various plant-produced isothiocyanates [49]. Deletion of the *saxA* gene in one of the gut microbes, *Pectobacterium carotivorum*, prevented this bacterium from being able to degrade plant material [38]. Whether these gut microbes are essential for the larvae to detoxify their host plant’s defense system and/or their possession of the detoxification enzymes to occupy this specific niche is not clear yet.

A third example of GBCs playing a role in detoxification is provided to blood-feeding dipterans, which also encounter toxic substances. Mosquitos are confronted by various toxic oxidants from blood, especially reactive oxygen species (ROS) and reactive nitrogen oxygen species (RNOS) [50,51]. A mixture of gut microbes, such as *Klebsiella*, *Serratia*, *Enterobacter*, and *Pseudomonas*, isolated from the blood-feeding insects *Anopheles gambiae* and *Lutzomyia longipalpis*, degraded toxic oxidants by producing microbial antioxidants [23,50]. However, GBCs seem not to be essential for all mosquitos to survive on blood meal. Reduction of the GBCs in *Aedes aegypti* females by different antibiotics had no effect on their survival [52]. However, it reduced the lysis of the red blood cells, digestion of blood proteins, and egg production in this species [52]. This suggests that at least some mosquitos possess mechanisms to overcome the toxic oxidants in blood meals by themselves. Other than the bacteria, which catabolize the oxidants, several mosquitos tolerate oxidants by producing a peritrophic matrix structure in their gut after the ingestion of a blood meal, such as *A. aegypti*, *An. gambiae*, *Anopheles stephensi*, *Anopheles labranchiae*, and *Culex tarsalis* [53,54]. This membrane separates the epithelium cells from ingested blood and reduces damage to other organs [53,54]. In waste-feeding Diptera, GBCs affect host performance. Studies in *Musca domestica* showed that the removal of GBCs reduced the growth and development of this fly species [55,56]. The examples above show that GBCs can be essential to exploring specific habitats and food sources for some dipteran species, such as the olive flies or the chironomid larvae, or in the case of *M. domestica*, affect their growth and development. In other dipterans, it is not so clear how much the insect depends on the GBCs. These dipteran species may have evolved their own strategies to cope with toxic diets and habitats.

## 3. Effects of Diet on Differences in Gut Microbial Community Composition among Dipteran Species

Considering the diversity of food sources consumed by dipterans, it can be expected that GBCs are equally diverse and differ among fly species, depending on their diet. A recent review comprising 21 insect orders showed that, overall, GBC diversity increased from blood-sucking insects via herbivores and carnivores to omnivores [57]. That may be due to the nearly sterile diets that blood-sucking insects consume, whereas omnivores have to handle a wide spectrum of food sources that themselves may contain rich microbial communities [57].

**Figure 1:**
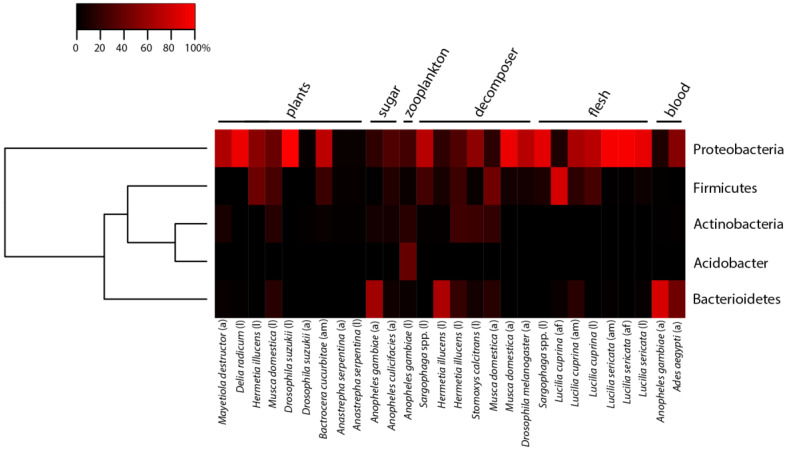
Gut bacterial community composition in Diptera that is based on the studies in Table 1. Heatmap of gut bacterial composition in dipteran species feeding on various diets (plants, sugar, zooplankton, decomposer, flesh, or blood). Relative abundance (percentage of total) of bacteria is indicated at a phylum level. The life stages of the hosts (a: adult, l: larvae) and their sex (m: male, f: female) are indicated next to the species name.

The question we address here is whether we observe a diet-specific pattern in GBCs in a subset of Diptera with different food sources. We addressed this question by using published GBC datasets on the bacterial phylum level (Figure 1) and more in detail on the genus level (Table S1). We searched the scientific literature for datasets of Diptera and only kept studies that analyzed insects fed on natural food sources (except for *An. gambiae* and *Anopheles culicifacies*) and used culture-independent methods to analyze GBCs. We tried to cover a broad range of food sources and to include different families. The papers were also screened for GBC identification on the genus level. On the basis of our criteria, we selected 16 different studies on 15 different dipteran species and 27 different samples (Table 1). The diets of the dipterans ranged from plant material and sugar to zooplankton, waste, vertebrate tissues, and blood (Table 1). The data also included different life stages, partly on single species (five studies, seven species) and data on a single life stage offered natural and artificial diets (two studies, two species). Additionally, we extracted data on the presence of bacterial genera where available (Table S1). To compare the GBCs, we generated a heatmap with the gplots package 3.0.1.2 [58] in R 3.6.2 [59]. A one-way hierarchical clustering heatmap was generated using the relative abundance of bacterial phyla. Dipteran species were manually sorted according to their food sources. This allowed us to identify similarities and differences in GBCs among the tested dipteran species on the level of bacterial phyla.

Overall, the dipteran GBCs reported in these 16 studies were dominated by Proteobacteria, Firmicutes, and Bacterioidetes and did not cluster strictly by diet (Figure 1). Within the phylum Proteobacteria, the Gammaproteobacteria were the most abundant inhabitants of the included insect guts, with the taxa *Providencia*, *Morganella*, *Pseudomonas*, and *Serratia* occurring in nearly all species (Table S1) [61,74,75]. *Providencia* is known to enable xylan digestion, a common compound of cell walls [76]. The ability to digest xylan is particularly important for arthropod decomposers, especially those living in dead trees or in litter containing a lot of bark. *Morganella* on the other hand is mainly known as a human pathogen [77]. Even through *Morganella* is lethal to the Mexican fruit fly (*Anastrepha ludens* Loew), it occurs in this species as well as the gut of many other fly species [78,79,80], where its function is unknown. Presumably *Morganella* is taken up with food. *Pseudomonas* sp. are also commonly found in dipteran guts (Table S1) [50,60,61,65,73,74]. Several *Pseudomonas* strains are able to protect their hosts from endopathogenic fungi by producing antimicrobial substances [81]. Pathogenic microorganisms are omnipresent, which makes *Pseudomonas* an important GBC member with potential benefits to their host [82]. The effects of *Serratia* strains, which are frequently found in dipteran guts, can range from lethal to essential in their hosts. Some strains have a strong entomopathogenic effect through the production of chitinases and proteinases, for instance for weevils or *Drosophila* [83,84]. Other strains improve host nutritional status by producing amino acids, or defend their host by enhancing host immunity [85,86]. Both functions enable their hosts to live under challenging conditions.

We expected that species with similar diets also have similar gut microbiome communities. Figure 1 shows that of the dipteran species we considered, only flesh-feeding species had rather uniform GBCs at the phylum level. On lower taxonomic levels, several bacterial genera, such as *Carnobacterium*, *Psychrobacillus*, or *Empedobacter*, were almost exclusively detected in the carnivorous insects studied herein (Table S1). Carnivores and insects feeding on waste also share several microbial taxa (Table S1). *Proteus* seems to be a diet-specific genus of Diptera that feeds on flesh and waste (Table S1); it dominated in the larvae of the flesh flies we considered here, and it was also present in their diet and their parasitoids (*Nasonia vitripennis* (Walker) and *N. giraulti* (Darling)) [87]. Some *Proteus* strains, for instance *Proteus vulgaris* and *Proteus mirabilis*, produce antimicrobial compounds, that are active against *Echerichia coli* and *Staphylococcus aureus* [75,88]. This may be beneficial to the hosts, as both waste and flesh contain a high proportion of microbes that can also be pathogenic to the insects. Hosting *Proteus* strains may thus protect these flies against these pathogens, which they commonly encounter in their diet [75].

On the basis of the published data we considered here, we found no confirmation that the GBC composition was mainly driven by host diet. We assume that some Diptera possess mainly generic gut microbes, and only very few bacterial genera are associated with a specific diet. This makes it difficult to extrapolate putative functions with regards to diet specialization on the basis of GBC composition. Although 16S rRNA amplicon sequencing enables culture-independent identification of bacteria, it does not distinguish between living and dead bacteria nor resident and transient species. This lack of information prohibits us to draw strong conclusions on the relation between GBCs and diet specialization. Variation in sample collection, such as from dissected guts or surface-sterilized insects, and data processing may be additional sources of variation beyond our control, which may have prohibited us from finding a clearer signature of food source in GBCs.

## 4. Additional Sources of Variation in Dipteran GBC

A second hypothesis we tested is whether GBCs vary per species. On the phylum level, we found no evidence that host species determined the GBC. On the genus level, *Anopheles gambiae* harbors a few species-specific bacteria in its GBC (Table S1). For instance, *Elisabethkingia* was only found in *Anopheles*, independent of the life cycle stage and food source (Table S1) [89]. A reason that the GBCs do not cluster per species in Figure 1 may be that dipterans are confronted with a large number of variable factors in their life. First, as holometabolic insects, they pass through several distinct life stages. In many cases, transitions from one life stage into another come along with shifts in diet, habitat, and behavior [90,91,92]. These changes may result in GBC transitions within a life cycle [93]. Moreover, the GBC data used for the analyses may also vary because they are from different origins (natural or lab cultures), or are taken from different populations. In addition, GBCs are influenced by interactions within the GBC and with other microorganisms, such as microbes on the food or the aforementioned endosymbionts. Each of these factors might prohibit that we find strong indications for a stable, species- and/or diet-specific GBC in the dipteran species analyzed.

### 4.1. Variations in GBCs during the Life Cycle

Throughout their life, dipterans come in contact with a large diversity of microbes. A subset of these microbes can be included in their guts. These microbes can be acquired via the parents (vertical transmission) or via the environment (horizontal transmission) [94,95]. Vertical transmission ensures that symbiotic microbes that are essential for survival are successfully transferred. In other words, the progeny benefits from “inheriting” advantageous microbe–host interactions from their parents, which have become established over a long period of time. Moreover, habitat and/or diet changes within a life cycle are common in the Diptera. Microbes that are no longer relevant can be removed from the community, thereby preventing their use of resources without providing benefits. Horizontal transmission may be advantageous when there are strong shifts in habitats or feeding habits between life stages. GBCs may result from a combination of horizontal and vertical transmission [96]. Due to the large numbers and diversity of microbes and the different transmission possibilities (vertical and horizontal), dipteran GBCs can be very diverse. To cover their needs, Diptera maintain specific conditions in their digestive tract that can “filter out” specific bacteria.

**Figure 2:**
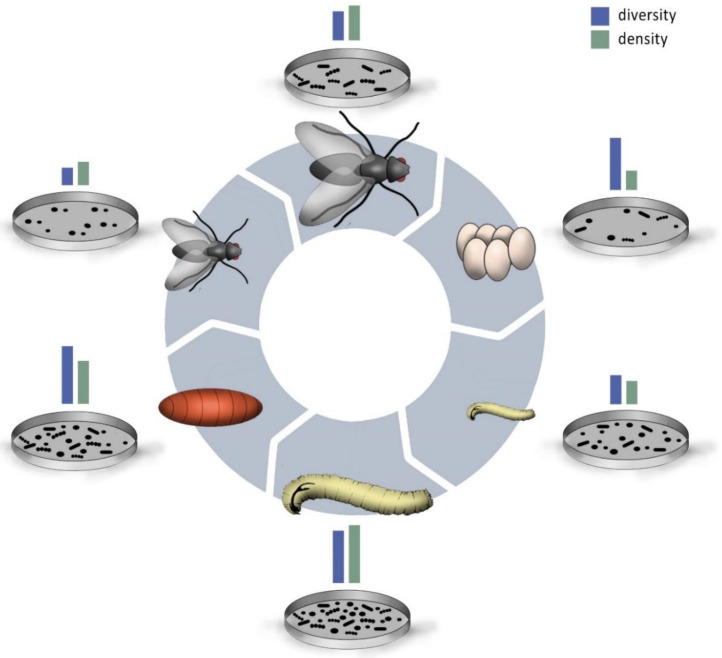
Schematic representation of the changes in gut bacterial density and diversity over the course of the life cycle including egg, larval, pupal, and adult life stages that is based on the current literature reporting on seven species (Table S1). In green: relative gut bacterial community (GBC) density, in blue: relative GBC diversity. Relative changes in gut bacterial diversity and densities are represented by the different shapes and numbers of microbes in a Petri dish. (Picture: Jennifer Gabriel).

Vertical transfer of GBCs from adults to eggs was found in tephritid fruit flies and *A. aegypti* [97,98,99]. The microbial community on the egg surface was mostly derived from the adult’s gastrointestinal tracts. The adults transfer the microbes by smearing feces on the egg shells after oviposition [37]. The few studies there have shown that dipteran eggs have a low density, but a high diversity of bacteria (Figure 2) [100,101]. Some freshly hatched larvae consume the egg shell and thus inoculate their guts with these microbes [93]. The tsetse fly, a blood-feeding species, directly transfers obligate symbionts to the larvae. The larvae hatch in the mother, where they are nourished with special glands [102]. Beside proteins, lipids, and amino acids, the “mother milk” also contains bacteria [103]. In this way, the parental GBC co-determines the GBC of their offspring. However, only a subset of the parental gut microbes may be able to establish in the offspring. For example, in the eggs of a chironomid species, the dominant bacteria belong to Proteobacteria, whereas Firmicutes dominate in larvae [37]. This might be due to morphological and hormonal changes, as well as drastic changes in diets and environmental conditions between their life stages [37]. In the case of mosquitos, the larvae live in the water where they feed on algae and zooplankton (Figure 1) [104,105]. Male and female adults feed on nectar or honeydew, whereas only females of most species need a blood meal to produce eggs [106].

In addition, molting and pupation may be barriers to the smooth transition of GBCs to the next life stage. During the larval stage, dipterans pass through two to five molts [107]. The cuticles of the fore- and hindgut are regenerated after each molting process, meaning that the old ones are shed with a large part of the resident microbiome. Consequently, gut microbial composition and abundance vary among larval instars [108,109]. Total bacterial abundance can increase throughout the larval stages and peak in the last instar, just before the last defecation (Figure 2) [108,110,111]. There may be a positive correlation of larval stage and bacterial abundance. Larvae in later instars have probably been confronted with a wide variety of microbes, much more so than younger larvae.

At the end of the last instar, microbial abundance can strongly decrease. In this stage, the majority of gut microbes and digested food material are removed with defecation and additional excretion processes [111]. The microbial communities may be shed to allow metamorphosis to take place.

Metamorphosis is initiated by specific physiological and environmental conditions (body size, mass, specific hormone ratio) [112,113,114,115]. During metamorphosis, dipteran larvae replace nearly all their organs and tissues [107].

Most of the larval gut cells (fore- and hindgut) are released into the body cavity, and new structures are formed from cells of the fore- and hindgut [93,107,111]. In this process, the larval midgut is almost completely recycled. Larval midgut cells and GBCs are sloughed and degenerated. The remaining larval cells, remnant nutrition, and possibly also some surviving gut microbes remain in the lumen, and in several fly species may form the meconium [97,116,117]. In the parasitic spotted flesh fly *Wohlfahrtia magnifica* (Schiner), the abundance of gram-negative bacteria was particularly reduced after pupation [75]. This decrease correlated with the expression of several antimicrobial protein genes. These proteins are predicted to inhibit bacterial growth over metamorphosis, which may be another reason for the low bacterial abundances commonly found in pupae and freshly emerged adults [50,93,111]. Importantly, pupae do not feed, which excludes diet as a source of bacterial uptake. Even through eggs and pupae are non-feeding stages, the two life stages do not necessarily share the same microbial community patterns. In blow flies, Actinobacteria and Acidobacteria prevailed in eggs, whereas Flavobacteriaceae and Bacilliales dominated in pupae [74]. This means that over the entire life cycle, bacterial communities can be very variable. A few bacteria seem to be present in all life stages. A study about GBCs in *Drosophila suzukii* found a similar abundance of a few bacteria in larvae and adults, such as *Tatumella punctate*; however, the abundance of several other gut bacteria also differed in both life stages [71]. We assume that the GBCs follow the needs of each life stage to a certain extent, rather than being species-specific.

Freshly eclosed adults of *A. gambiae* have a nearly sterile gut, as most of the remaining gut microbes are removed with the meconium directly after eclosion [50]. Over the adults’ life time, the density of microbial communities can increases with age (Figure 2) [50,93]. While the microbes increase in numbers, the GBC composition can also change. *Lactobacillus* is dominant in young *Drosophila* flies, and Acetobacteria in older flies [93]. This confirms that GBCs may be mainly stage-specific instead of species-specific in some species. Stage-specific microbes may confer specific functions. The bacterium *Proteus vulgaris* was predominant in larvae and strongly reduced in several adult dipteran species [72]. It was suggested that *Proteus vulgaris* is involved in the digestion of larval food sources, survival in the larval environment, or metamorphosis [72,75]. Both switches in diets between life stages (which may require different GBCs) and new environments/diets (which present a large resource for new microbes) may generate stage-specific instead of species-specific GBCs in some cases. The inclusion of microbes from new environments/diets upon changing life stage depends on microbial availability and microbial resistance against the defense barriers of the host insect [108]. Furthermore, the “new” microbes have to compete with the established GBCs. Due to the fact that changes in life stages co-occur with diet shifts, the effects of these two factors on GBCs are hard to disentangle.

### 4.2. Effect of Environment on Dipteran GBCs

Diptera are masters of adaptation. They exploit habitats that contain toxic organic or inorganic compounds or are low in oxygen. Within their lifetime, they may change from aquatic to terrestrial biomes and feed on different food sources. Furthermore, several Diptera, such as *Drosophila*, *Musca*, and *Delia* spp., can be easily reared under laboratory conditions. Each of these environmental factors alone, or in combination, may affect their GBCs.

Dipteran species live in biomes with contrasting environmental conditions. These range from (largely) anaerobic conditions, under water or in the soil, to aerobic aboveground terrestrial ecosystems where high temperatures may be an issue [118,119,120]. Such strong contrasts may shape GBC compositions. In most other insect orders, GBCs of terrestrial insects were richer in aerobic microbes than those of aquatic insects. This may be also true in Diptera [57], where facultative anaerobic microbes were found in the guts of aquatic stages in Culicidae [98]. Moreover, different life stages of one species can live in different habitats. Mosquitos start their life in water and move to terrestrial habitats in the adult stage [121]. In *A. aegypti*, microbial diversity and abundance changed significantly between both stages. The microbial diversity declined from larvae (74 operational taxonomic unit (OTUs)) to adults feeding on sugar (39 OTUs) [98]. A blood meal reduced the diversity even more to 22 OTUs. The larval GBCs were dominated by *Leucobacter* and *Microbacterium* (Actinobacteria), which were nearly absent in the adults. Adult GBCs were dominated by *Pseudomonas*, *Paenibacillus*, *Aeromonas*, *Aquitalea*, and *Stenotrophomonas* (Gamma- and Betaproteobacteria, Firmicutes). These groups were only found in low numbers in the larvae. The GBCs of females that had a blood meal were dominated by the two bacteria strains *Chryseobacterium* and *Delfia* (both Betaproteobacteria) [98]. Several of these strains turned out to be essential for the larvae to reach the adult stage. Whereas axenic larvae failed to develop beyond the first instar, adding *Acinetobacter*, *Paenibacillus*, *Aeromona*, *Aquitalea*, or *Chryseobacterium* to the axenic larvae enabled them to reach the adult stage [98]. Some of these bacteria produce signal molecules that regulate growth and metabolism, helping them to reach the adult stage [122]. This may explain why the GBC abundance in *Drosophila melanogaster*, *Chrysomya megacephala*, *Bactrocera dorsalis*, and *An. gambiae* decreased from the larval to the adult stage [50,100,101,123].

Another variable factor that dipterans have to handle is temperature. This abiotic factor not only determines developmental time in Diptera [124], but it may also affect GBCs. For instance, both *Wolbachia* and *Acetobacter* live inside *D. melanogaster*. When the host developed at colder temperatures, *Wolbachia* predominated, whereas at higher temperatures *Acetobacter* was more important [125]. This may also be true for other gut bacteria. GBCs may also differ over the seasons, which strongly correlate with shifts in temperature and diets. In green bottle fly GBCs (Calliphoridae), *Staphylococcus* was dominant in spring; *Ignatzschineria* in summer; and *Vagococcus*, *Dysgonomonas*, and an unclassified Acetobacteraceae in autumn [126]. The GBC diversity tended to increase from spring (24 OTUs) to autumn (93 OTUs) in these flies. Wei et al. [126] suggested that changes in climatic conditions were the main cause of seasonal variations in fly-associated bacterial communities. However, seasonal changes also come with differences in the environmental microbial communities, including those on food resources. More studies are necessary to show whether GBC variations over seasons are a general pattern in Diptera. Specific manipulative experiments can disentangle the effect of temperature from other factors that change with the season, such as rainfall patterns, UV/sunlight radiation, day length, and the availability of food resources. Such knowledge may also help to predict impacts of global change on dipteran GBCs. On the one hand, GBCs may affect the availability of the host to adapt to global changes. On the other hand, global change may also affect the occurrence of microbes in the host’s environment and diet [127].

In most cases, both food source and habitat conditions are markedly different between natural and laboratory systems. In laboratory cultures, biotic and abiotic factors vary very little because the rearing conditions are optimized to produce as many individuals as possible [128]. The absence of interactions may lift natural selection pressures, such as enemies, competition with other species, and fluctuating environmental conditions, which normally would shape GBCs. In particular, the lack of microbial cross-infestations from other species and/or different diets may restrict fly-associated microbial community development in laboratory cultures. Laboratory-reared *Drosophila* flies indeed possess a less diverse gut microbiome, compared to flies caught in the wild (2.4–5.3 times more OTUs). Moreover, the GBC composition differed; in GBCs of lab-reared flies, *Acetobacter* and *Lactobacillus* dominated, whereas in conspecifics from the field, Proteobacteria in particular were found [93,96,100,129,130]. In contrast, GBCs of wild and laboratory *An. gambiae* strains were rather similar [50]. The same was true for housefly GBCs; essentially the same bacteria species were isolated from the digestive tract of field-collected and laboratory-reared house flies, independent of life stage and collection year [56,131]. On the basis of these few examples, it is too early to conclude whether the GBCs of lab-reared strains are consistently different from conspecifics in their natural habitat. More specific analysis comparing GBCs of lab-reared with field-caught dipterans are needed to address this question.

### 4.3. Interactions with Other Microorganisms

Apart from the internal and external factors discussed in the previous sections, GBCs may also be affected by interactions with other microbes. Several dipterans vector microbial human pathogens, which may interface with GBCs. In addition, gut microbes may compete among each other as well as with other microbes colonizing their host, such as endosymbionts. Such “host-internal” microbial interactions can both affect and be affected by GBC composition.

Dipterans are well-known vectors of a wide variety of vertebrate diseases, such as malaria and yellow fever [132,133]. The pathogens are mainly bacteria, viruses, or protists, here collectively referred to as human pathogens. Human pathogens interact in different manners with the GBCs of their vectors. First, human pathogens can decrease abundance of particular microbes, thereby altering GBC compositions in some Diptera. For example, the pathogen *Leishmania mexicana* is vectored by sand flies. Its presence in the fly decreases the microbial richness in the insect’s GBC [134]. Several Pseudomonadaceae were reduced in their abundance, whereas Acetobacteraceae became dominant with increasing pathogen densities [134]. Second, dipteran GBCs can affect the development of human pathogens [135]. For many human pathogens, it is essential to replicate or to go through several steps of differentiation in the vector before they can infect humans. Both inhibitory and beneficial effects are reported. GBCs of the mosquitos *An. stephensi* and *An. albimanus* inhibited the development of the malaria pathogen *Plasmodium* through the activation of general immune system responses in the guts of these mosquitos [136,137,138]. The natural common gut bacterium *Enterobacter* produces reactive oxygen species in the midgut of the mosquito *An. gambiae*. The reactive oxygen directly inhibits the development of the malaria pathogen *Plasmodium* [139]. This indicates that GBCs of the vectors can affect the transmission of human pathogens through the inhibition of human pathogen development in their vector. Human pathogens can also benefit from the vector’s GBCs. GBCs of sand flies improved the growth and development of infective stages in the pathogen *Leishmania infantum* [23]. Experimental applications of antibiotics to *L. infantum*-infected sand flies reduced the replication and the development to infectious stages in the vector’s gut, without affecting sand fly fitness [134]. Thus, GBCs can be critical factors for the survival of human pathogens, their differentiation to infective stages, and disease transmission. On the basis of their impact on pathogen life cycles, GBCs provide a potential as biological control for vector transmitted diseases.

Besides human pathogens, other GBC members can also affect GBC composition. Interactions between different GBC members can influence gut microbial co-occurrence in a positive, negative, or neutral way. Neutral co-occurrence was the most commonly observed interaction within gut microbial taxa in *D. melanogaster*, except for the three following groups [70]. Xanthomonadaceae showed positive co-occurrence effects mainly with taxonomically related strains. *Enterococcus* and *Staphylococcus aureus* showed negative co-occurrence effects mainly with non-closely related strains. Overall, taxa that are interacting with many other bacteria were not the most common ones. It seems that the dominant taxa are not the mayor players in structuring GBCs in *D. melanogaster* [70]. To understand interactions of GBCs with each other and their hosts, we have to gain a better understanding of gut microbe communications and to identify regulatory mediators in GBCs. This could be achieved by analyzing meta-transcriptomes of GBCs challenged with different interaction partners.

## 5. Conclusions and Future Perspectives

In this review, we explored factors that may determine the composition of GBCs in Diptera. We considered species-related effects, food sources, environmental conditions, life history, and interactions with other microorganisms. On the basis of our findings, diet can partly explain differences in GBCs in the species we could obtain data on, but this applies mostly to species feeding on vertebrate tissues and blood. Changes in food sources are often linked to different life stages. Life cycle transitions may have a strong impact on GBCs due to drastic morphological changes during metamorphosis. Likely, this prevents the development of a strictly species-specific GBC in some dipterans.

Our review revealed several factors that limit our ability to understand the role of GBCs in Diptera. The main limiting factor is the low number of studies. Even for the order of Diptera, which contains over 150,000 species, the GBCs of only a handful of species have been studied in sufficient detail. Moreover, the studies we analyzed focused mainly on species with economic impact or vectors of human pathogens. More dipteran species, and within each species more life stages, must be analyzed before we can draw firm conclusions how GBC patterns relate to dipteran diversity. A second limiting factor is the classification of bacteria. In general, bacteria are differentiated according to their genetic and phenotypic similarity [140]. However, genetic similarity is often based on a single gene, the 16S rRNA, for both the commonly used OTU and amplicon sequence variant (ASV), which does not necessarily reflect similarity at the whole-genome level. In addition, horizontal gene transfer and mutations occur relatively frequently in bacteria. Such minor changes in the genome can have strong effects on the metabolism. Bacteria with genetic and phenotypic similarity but functional differences are therefore usually divided into separate strains. It is possible that the same ASVs occur in the guts of different insects, but that these ASVs have different functions. This “hidden” level of bacterial diversity prohibits firm statements on bacterial functional diversity.

Despite the increased interest of GBCs in macroorganisms, a large gap remains, namely, the function of the entire GBC versus single members therein. Massive parallel sequencing approaches combined with bioinformatics, collectively called metagenomics, allow us to compare GBCs and identify differences in compositions among species, life stages, and diets in much more detail. However, such comparisons only reveal who is there, and not which function the GBC, or single species therein, might have. Metatranscriptomic analyses identify which genes are up- or down-regulated in a GBC, for example, in response to toxins or environmental changes. This helps to identify possible functions, for instance a main detoxification enzyme, present in the GBC. However, metagenomics and -transcriptomic analyses only generate hypotheses on which function could be important. Preferably, they should be combined with manipulative experiments, knocking out a specific function after which insect performance is also assessed. This approach could also help to identify potential targets for pest management. Targeting stage-specific gut microbes seems a promising control strategy to interfere with the insect’s life cycle. This may prohibit the development of the critical stage or sex, for example, the emergence of female mosquitos that vector human pathogens. In order to develop such strategies, more specific knowledge about GBCs and its role in each of the host’s life cycle stages are needed.

In addition, we lack understanding of interactions within GBCs or between GBCs and their host. Within GBCs, many interactions may occur; among bacterial species competing for a niche, and among bacteria, fungi, and viruses. To better understand these processes, we would recommend the use of synthetic GBCs that could be fed to sterile insects [141]. These insects could be exposed to different conditions, and the resulting GBCs as well as the host performance could be measured. This would allow us to identify drivers and passengers in GBCs or help to identify specific functions provided by the host. We can draw on tools from biodiversity science and restoration ecology to analyze GBC interaction webs and identify key organisms [142]. Ecosystem service analyses may predict connections among GBC members and identify their functional roles [143]. Possible features to use in GBC models are nutritional data, detoxification mechanisms, and the ability to produce antibiotics by single community members. These data can be obtained from metagenomic or -transcriptomic analyses. The exact signals involved in microbe–microbe communication and how they affect GBC structures are still largely unknown. Integrating metabolomic analyses and molecular diagnostic approaches (e.g., Raman spectroscopy) would be a possible approach to fill this gap.

By studying dipteran GBCs, we will also increase understanding the ecology of the insect hosts and their adaptation to recalcitrant diets and habitats. We recognized that only very few studies report on GBCs in Diptera, particularly in relation with food sources. This makes it very difficult to draw conclusions about diet specificity of GBCs. In order to do so, we would need phylogenetically controlled GBC composition data, for instance screening a single genus with multiple feeding strategies or specialization levels. In addition, having multiple replicates of the same feeding strategies over distant lineages, such as Culicidae vs. Tabanidae, would improve our knowledge on the role of GBCs in habitat adaptations. The same is true for the dynamics of GBCs during the life cycle. Only a handful of studies analyze the GBCs of life cycle stages; studies comparing the fate of GBCs over life cycles of several species are even rarer. More research is needed, especially experimental studies, to understand host–GBC interactions and dynamics within GBCs. Utilizing approaches from other disciplines facilitates the development of new concepts and will help to test current hypotheses on the function of dipteran GBCs.

## Figures and Tables

**Table 1:** Food sources in Diptera. Overview of food sources in different dipteran species, life stages, and corresponding studies, which were used for detecting diet-specific patterns in dipteran gut microbial communities.

Species	Life Stage	Food Source	Origin	Method	Reference
*Mayetiola destructor*	Adult	Plants (leaves)	Laboratory	16S rRNA (454-pyrosequencing)	[60]
*Delia radicum*	Larvae	Plants (roots)	Laboratory	16S rRNA (Ion Torrent)	[61]
*Bactrocera cucurbitae*	Adult	Plants (fruits)	Natural	16S rRNA (ABI)	[62]
*Anastrepha serpentina*	Adult	Plants (fruits)	Natural	16S rRNA (454-pyrosequencing)	[63]
	Larvae	Plants (fruits)	Natural	16S rRNA (454-pyrosequencing)	
*Hermetia illucens*	Larvae	Plants (seeds)	Laboratory	16S rRNA (454-pyrosequencing)	[64]
		Decomposers (omnivore)			
		Decomposers (animal)			
*Anopheles gambiae*	Adult	Sugar solution	Laboratory	16S rRNA (454-pyrosequencing)	[50]
		Blood			
	Larvae	zooplankton			
*Anopheles culicifacies*	Adult	Sugar solution	Laboratory	16S rRNA (454-pyrosequencing)	[65]
*Ades aegypti*	Adult	Blood	Laboratory	16S rRNA (Illumina)	[66]
*Musca domestica*	Adult	Decomposers (omnivore)	Natural	16S rRNA (Illumina, ABI)	[67,68]
	Larvae	Plants (seeds)	Laboratory	16S rRNA (Illumina)	[69]
*Drosophila melanogaster*	Adult	Decomposers (plants)	Natural	16S rRNA (Illumina)	[70]
*Drosophila suzukii*	Adult	Plant (fruits)	Natural	16S rRNA (Illumina)	[71]
	Larvae	Plants (fruits)	Natural	16S rRNA (Illumina)	
*Sargophaga* spp.	Adult	Decomposer (omnivores)	Natural	16S rRNA (ABI)	[72]
	Larvae	Flesh			
*Stomoxys calcitrans*	Larvae	Decomposer (omnivore)	Natural	16S rRNA (454-pyrosequencing)	[73]
*Lucilia cuprina*	Adult	Flesh	Laboratory	16S rRNA (454-pyrosequencing)	[74]
	Larvae	Flesh			
*Lucilia sericata*	Adult Larvae	Flesh Flesh	Laboratory	16S rRNA (454-pyrosequencing)	[74]

## Supplementary Materials

The following are available online at https://www.mdpi.com/2075-4450/11/8/543/s1, Table S1: Presence or absence of gut bacteria on the genus level in selected dipteran species, life stages and food source, Table S2: Density and diversity of gut microbes in seven Diptera species.
